# Water-Mediated Recognition of Simple Alkyl Chains by Heart-Type Fatty-Acid-Binding Protein**

**DOI:** 10.1002/anie.201409830

**Published:** 2014-12-09

**Authors:** Shigeru Matsuoka, Shigeru Sugiyama, Daisuke Matsuoka, Mika Hirose, Sébastien Lethu, Hikaru Ano, Toshiaki Hara, Osamu Ichihara, S Roy Kimura, Satoshi Murakami, Hanako Ishida, Eiichi Mizohata, Tsuyoshi Inoue, Michio Murata

**Affiliations:** JST-ERATO and Graduate School of Science, Osaka UniversityMachikaneyama, Toyonaka, Osaka 560-0043 (Japan); Project Research Centre for Fundamental Science, Osaka UniversityMachikaneyama, Toyonaka, Osaka 560-0043 (Japan); Schrödinger K. K., 17F Marunouchi Trust Tower North1-8-1 Marunouchi, Chiyoda-ku, Tokyo 100-0005 (Japan); Graduate School of Biosci./Biotech., Tokyo Institute of Technology4259-J2-17 Nagatsuta, Midori-ku, Yokohama 226-8503 (Japan); Graduate School of Engineering, Osaka UniversityYamadaoka, Suita 565-0871 (Japan)

**Keywords:** fatty acids, molecular dynamics, molecular evolution, structural biology, water clusters

## Abstract

Long-chain fatty acids (FAs) with low water solubility require fatty-acid-binding proteins (FABPs) to transport them from cytoplasm to the mitochondria for energy production. However, the precise mechanism by which these proteins recognize the various lengths of simple alkyl chains of FAs with similar high affinity remains unknown. To address this question, we employed a newly developed calorimetric method for comprehensively evaluating the affinity of FAs, sub-Angstrom X-ray crystallography to accurately determine their 3D structure, and energy calculations of the coexisting water molecules using the computer program WaterMap. Our results clearly showed that the heart-type FABP (FABP3) preferentially incorporates a U-shaped FA of C10–C18 using a lipid-compatible water cluster, and excludes longer FAs using a chain-length-limiting water cluster. These mechanisms could help us gain a general understanding of how proteins recognize diverse lipids with different chain lengths.

For energy production in the skeletal and heart muscle,[1] the efficient cytosolic delivery of fuel such as long-chain fatty acids (LCFAs) is crucial. Mitochondrial metabolism prefers fatty acids (FAs) of a certain range of chain length. Thus, specific transporter and carrier proteins of the “fuel” FAs have been created as exemplified by the fatty-acid-binding proteins (FABPs).[2,3] However, it remains unclear how such proteins recognize FAs with flexible alkyl chains that do not exhibit a defined structure or noticeable electrostatic interactions. Moreover, this mechanism may provide an answer to the fundamental question of how lipid-binding proteins generally recognize the various lengths of simple alkyl chains. Here, we show that human heart-type FABP (FABP3) identifies FAs not by exact matching but by broad recognition of fundamental structural similarities among numerous FAs.

To date, more than 40 subtypes of FABPs have been identified,[4] most of which share a highly conserved three-dimensional structure.[3] As reported for FABP3, one LCFA molecule in a U-shape is accommodated in the binding cavity together with about 13 ordered water molecules.[5] A similar shape of the bound FA is seen with other subtypes, such as FABPs 4, 5, 7, and 8. In contrast, in the binding sites of nonspecific lipid transporters universally expressed from bacteria to humans,[6] the FAs are largely extended (Figure S1). These observations suggest that the U-shape conformation of bound FA is critical for the incorporation of FAs with different chain lengths into the binding site of FABP3 and the other FABPs, and raise an intriguing question as to how the proteins do this by using a rigid β-clam architecture and ordered water molecules in the pocket.

A previous study using radiolabeled FAs demonstrated that different FABPs had similar substrate selectivity; i.e., a higher affinity for longer FAs and a slight preference for unsaturated FAs.[7] However, these observations were made for LCFAs in aqueous media and might not accurately represent the case for the cellular environment. To overcome the low solubility of LCFAs and evaluate the binding of FA-FABP under cytoplasm-mimicking conditions, we designed a novel method using liposomes as vehicles for LCFAs. This is because cytosolic LCFAs are largely bound to hydrophobic sites such as lipid membranes, lipid droplets, and carrier proteins,[8] and FABP3 interacts directly with lipid bilayers upon FA transport.[9] We used isothermal titration calorimetry (ITC) to evaluate the binding affinity, and first recorded the thermograms for oleic acid (OA, C18:1), dimyristoylphosphatidylcholine (DMPC) liposomes, and OA/DMPC mixed liposomes. OA or DMPC liposomes showed no binding, whereas the OA-DMPC complex exhibited clear thermal responses (Figure 1).

**Figure 1:**
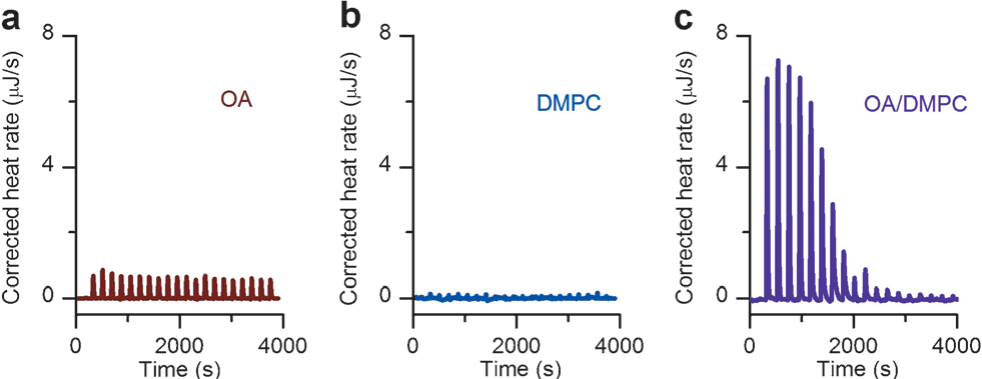
Isothermal titration calorimetry of oleic acid and DMPC liposomes. a) Oleic acid (OA, C18:1 n-9c) micelles; b) DMPC liposomes; and c) OA/DMPC (1/11 mol/mol) mixed liposomes were titrated into FABP3 solutions. Heat of binding was observed only when OA and DMPC were added as mixed liposomes.

Using the new ITC protocol, we succeeded in comprehensively evaluating FABP3’s affinity for 21 FAs varying in alkyl chain length and degree of unsaturation (Table S1a). In this study, the affinity of very long FAs such as C24:0 could be determined, which had been extremely difficult to do owing to their poor water solubility. Among saturated FAs (SFAs), FABP3 did not incorporate short-chain FA (C6:0), but did bind C7–C24 SFAs with *K*_d_ values of 1–50 μm, among which SFAs C10–C18 exhibited a 10-fold or even higher affinity (*K*_d_≈1 μm) than SFAs with shorter or longer chains (Figure 2 a). Unsaturated FAs with C18 chains had similar or slightly higher affinity for FABP3 than C18:0, but the selectivity for the number, position, or stereochemistry of C—C bonds was not apparent (Table S1a).

**Figure 2:**
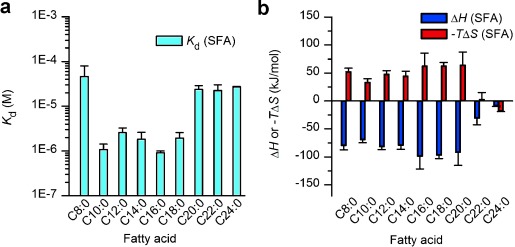
Fatty acid chain length preference of FABP3 as determined by liposome-mediated ITC. a) *K*_d_ values of FA/FABP3 complexes. b) Thermodynamic parameters. All experiments were carried out with FA/DMPC liposomes mixed at a 1:11 molar ratio. Error bars in (a) and (b) indicate the standard error (*n*=3).

To further confirm that chain-length selectivity is determined by the FABP3 structure, five SFAs (C10:0–C18:0) with higher affinity were cocrystallized with the protein and analyzed by X-ray diffraction at cryogenic temperature (Table S2), which resulted in their crystal structures with ultrahigh resolution (Figure 3 a–e and Figure 4). The 3D structure of the bound FAs thus obtained was significantly different from the previously reported one (Figure 3 f,g). By using these highly accurate FA structures, we attempted to examine the atomistic mechanism of FABP3’s preferential recognition of simple alkyl chains with a length of C10–C18. As reported by Sacchettini’s group,[5] these SFAs were firmly held at their carboxyl moieties by cationic residues (Figure 4 b). The side chains of two highly conserved hydrophobic amino acids (Phe16 and Ala75) closely interacted with FA molecules in the C7–C9 segment (Figure 4 b),[10] responsible for the higher affinity for FAs longer than C8. These spatial arrangements of FA and the side chains in the binding pocket were similarly observed at room temperature (Figure S2), at which the C12–C18 portion of the FAs became disordered as compared with the structures at low temperature. This segment-specific molecular recognition by FABP3 (Figure 4 c) should explain how the protein excludes short FAs; the C3–C6 portion of FA is only in contact with the water molecules (Cluster 1 in Figure 4 d and e).[5] The resultant entropy loss and the absence of stabilization by Phe16/Ala75 may account for the drastic drop in the binding affinity of C6 and shorter FAs. Furthermore, this mechanism should also be effective to reject structurally rigid or sterically bulk FA analogues such as retinoic/phytanic acids and oxylipins[11] (Table S1b).

**Figure 3:**
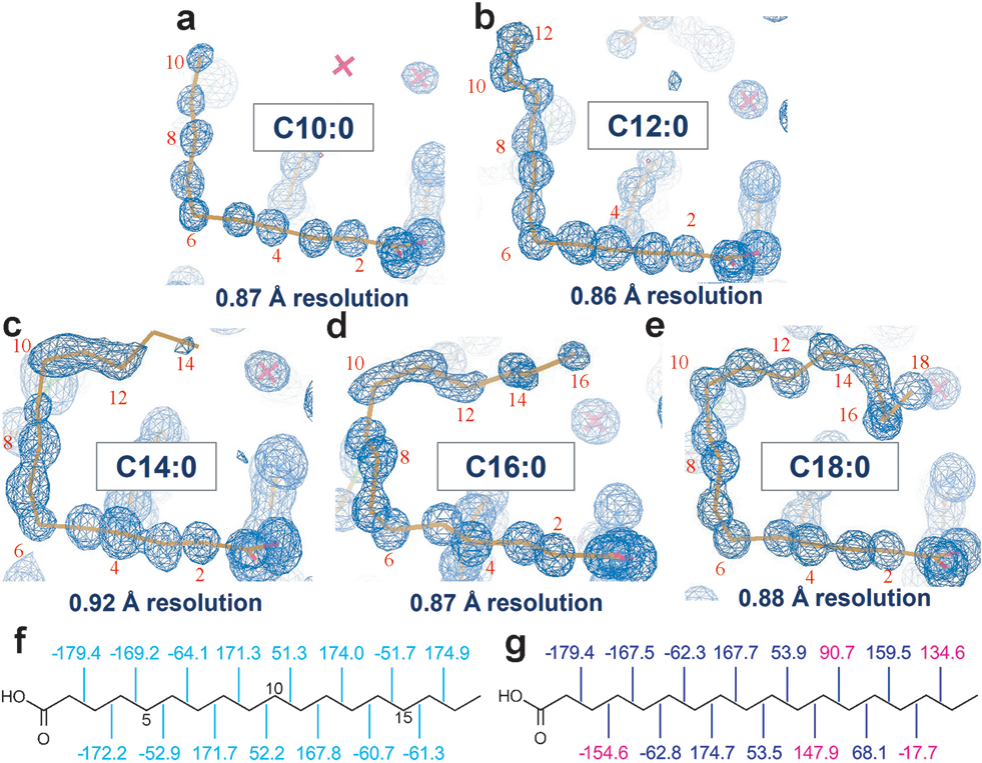
Electron density 2|*Fo* - *Fc*| map of five saturated FAs C10–C18 (a–e) bound to FABP3 at cryogenic temperature. As depicted, the resolution of each map was very high ranging between 0.86 Å and 0.92 Å. “X” denotes a water molecule; f) and g) dihedral angles of stearic acid bound to FABP3 in this study (PDB code: 3WVM) and a previous report (1HMT),[5] respectively, in which some dihedral angles (shown in red) differ from 60° or 180° by more than 25°.

**Figure 4:**
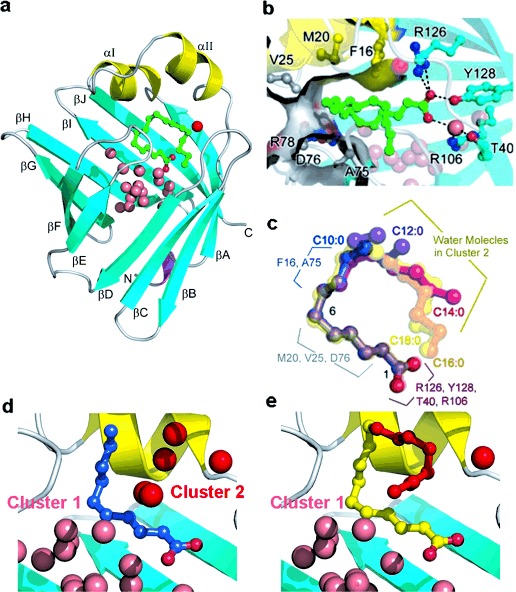
FABP3-SFA binding structures at cryogenic temperature. a) Crystal structure of human FABP3 obtained in this study (3WVM). b) The key intermolecular interaction sites in FABP3-FA interaction. c) Superposed main-chain structures of five FABP3-bound SFAs. d) and e) Binding pockets with C10:0 and C18:0, respectively, with the two clusters of water molecules.

In contrast to the structures of proteins that possess a flexible binding pocket to accommodate a wide variety of ligands such as cytochrome P450 and nuclear hormone receptors,[12] those of the FABP3 cavity incorporating different SFAs were virtually identical with the main chain RMSD of 0.13 Å (Figure S3) and the conformations of the C1–C10 portion of the FAs were also very similar (Figure 4 c). Instead, a cluster of ordered water molecules filled the cavity around alkyl chains of shorter FAs (Cluster 2 in Figure 4 d). These water molecules probably play a key role in the FABP3 preference for the U-shaped SFAs with a length of C10–C18 and also for unsaturated FAs with an inherent U-shape.

To elucidate the effect of water molecules on the SFA binding mechanism, the hydration states of the SFA/FABP3 complexes and their apo form were analyzed for molecular dynamics simulations. For this, the computational tool WaterMap,[13] which allows the estimation of the free energy associated with hydration, was applied to the X-ray crystallographic data. The molecular dynamics simulations predicted that, upon FA binding, these water molecules segregated into two distinct water clusters (Clusters 1 and 2 with significantly different energies) positioned in close agreement with the crystallographic data (Figure 4 d and Figure 5 b). The water molecules in Cluster 1, which were highly ordered, formed a stable hydrogen-bonding network[14] that was virtually identical in all five FA/FABP3 complexes (Figures 5 and S4b–S4f). On the other hand, Cluster 2 was confined to an unstable (high-energy) hydrophobic area surrounded by nonpolar amino acids and the FA hydrocarbon chain; a significant desolvation energy release was expected from the displacement of water molecules with an alkyl chain (Figures S4 and S5). It should be noted that the C18:0 chain almost exactly followed the high-energy hydration sites (Figure 5 c).

**Figure 5:**
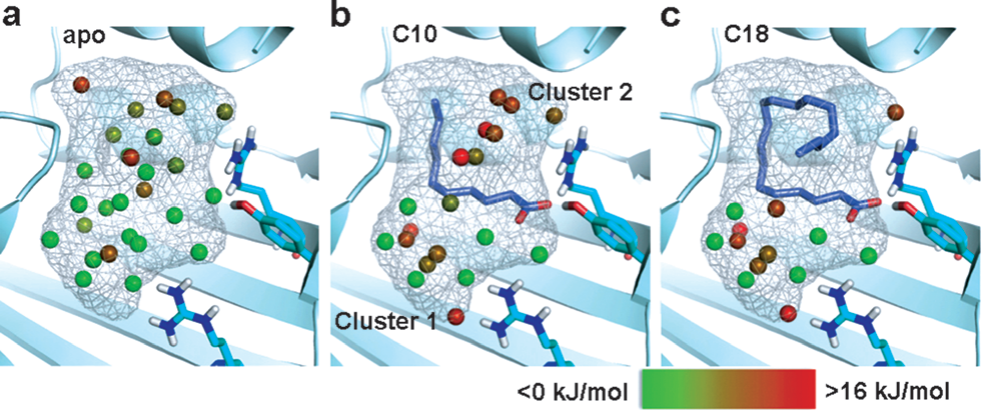
Hydration sites identified by WaterMap in the binding pocket of FABP3 cocrystallized with saturated FAs. Color gradation of the hydration sites is based on the free energy relative to bulk water; stable sites are shown in green and unstable sites in red. a) Hydration state of the apo binding site. b) Hydration state of FABP3 bound to C10:0. Note that water molecules are segregated into two clusters with distinct stability. c) The extended chain of C18:0 displaces unstable water molecules in cluster 2. Thermodynamic parameters are provided in Tables S3 and S4.

The ultrahigh-resolution X-ray cocrystallography and the WaterMap analysis confirm that the chain-length adaptability is supported by the disposition of the high-energy water molecules in Cluster 2. Their displacement with the C10–C18 alkyl chains leads to an enthalpy gain due to water purge, which is compensated by the entropy loss in the same process (Figures 2 b and S5). The lower affinity of the C20–C24 SFAs could be accounted for by the highly ordered water molecules, which contact with C18 FA at the top part of Cluster 1 (Figure 4 e). Further elongation of the FA tail should disrupt the stable water network, thus destabilizing the binding of longer FAs (Figure S4). Formation of this network is effected by the carboxylate at the FA head group, suggesting that a U-shaped FA in the binding pocket of FABP3, in which the tail comes close to the head, is the ultimate design for defining the preferred length of FA chains.

The highly flexible and nonpolar alkyl chains of lipids have prevented researchers from investigating the structure-based mechanism of their interactions with proteins. In this study, we focused on the rigid β-clam protein, FABP3. The room-temperature comprehensive ITC analysis and the low-temperature sub-Angstrom X-ray crystallography allowed us to examine FA-FABP3 interactions based on their thermodynamic profiles and high-resolution structures, respectively, for a series of FAs that varied in chain lengths. Using WaterMap and other simulations, we could correlate these two pieces of information to elucidate the structural basis for recognition of an alkyl chain by the protein, particularly the function of water clusters in incorporating a variety of “fuel” FAs. This promiscuous recognition might further explain the mechanism by which relatively rigid proteins accommodate lipids of different sizes, as was reported for START (Figure S6).[15] Moreover, flexible lipid-binding proteins, such as cytochrome P450,[16] and PPARs,[17] which are thought to select their ligands in an induced-fit manner (Table S5), might partly utilize this water-mediated mechanism to exhibit a promiscuous selectivity. The current strategy is potentially applicable to these flexible proteins, and could help provide a general understanding of how proteins recognize diverse lipids, possibly including those in cellular membranes.

The promiscuous binding by FABP3 is broad enough to include major FAs in mitochondrial energy metabolism (Figure S7). Phylogenetic studies suggest that FABP3 and other FABPs evolved from a common ancestor existed 1200–1000 million years ago when animals diverged from fungi and plants.[4,18] The structure of the ancestral protein with a fuel transfer “pocket” met high-energy requirements of animals and became well preserved in the contemporary FABP3 expressed in energy-demanding organs such as the heart and skeletal muscle, in which mitochondrial β-oxidation is active.
