# Supplementary material for: Water-Mediated Recognition of Simple Alkyl Chains by Heart-Type Fatty-Acid-Binding Protein
Source: Angew Chem Int Ed Engl. 2014 Dec 9;54(5):1508–11. doi: 10.1002/anie.201409830 (PMC4471613; doi:10.1002/anie.201409830)
Supplement: Supplementary file 1 [file anie0054-1508-sd1.pdf]

Angewandte Chemie
Eine Zeitschrift der Gesellschaft Deutscher Chemiker

Supporting Information

© Wiley-VCH 2015

69451 Weinheim, Germany

Water-Mediated Recognition of Simple Alkyl Chains by Heart-Type Fatty-Acid-Binding Protein**

*Shigeru Matsuoka, Shigeru Sugiyama, Daisuke Matsuoka, Mika Hirose, Sébastien Lethu, Hikaru Ano, Toshiaki Hara, Osamu Ichihara, S. Roy Kimura, Satoshi Murakami, Hanako Ishida, Eiichi Mizohata, Tsuyoshi Inoue, and Michio Murata**

anie_201409830_sm_miscellaneous_information.pdf

Supporting information

Water-Mediated Recognition of Simple Alkyl Chains by Heart-Type Fatty Acid-Binding Protein

Shigeru Matsuoka, Shigeru Sugiyama, Daisuke Matsuoka, Mika Hirose, Sébastien Lethu, Hikaru Ano, Toshiaki Hara, Osamu Ichihara, S. Roy Kimura, Satoshi Murakami, Hanako Ishida, Eiichi Mizohata, Tsuyoshi Inoue, and Michio Murata

Methods

Protein purification and delipidation. The gene of human heart-type fatty acid-binding protein (FABP3) was synthesized with an *Nde*1 site at the N-terminus and a BamH1 site at the C-terminus. The 399-bp fragment was ligated into the Nde1/BamH1-digested pET21a vector (Novagen). The expression vector (pET21a FABP3) was used to transform *Escherichia coli* BL21 (DE3) and the cell cultures were grown at 310 K. The transformed colonies was used to inoculate 2 L of 2×TY medium and the cell cultures were grown at 310 K. When the optical density of the medium reached 0.5 at 600 nm, FABP3 expression was induced by adding 0.5 mM (final concentration) isopropyl β-D-1-thiogalactopyranoside to the culture medium, and the cultivation was continued at 310 K for 3 h. The cells were harvested by centrifugation and then disrupted by sonication.

The sonicated medium was centrifuged at 100,000 g for 30 min at 277 K. Solid ammonium sulphate was added to the supernatant solution to give a final concentration of 50% saturation and the mixture was stirred for 1 h on ice. The suspension was centrifuged at 30,000 g for 30 min. FABP3 was mostly recovered in the supernatant. Additionally, solid ammonium sulphate was added to 70% saturation and the mixture was stirred for 1 h on ice. After centrifugation of the suspension for 30 min at 30,000 g, the supernatant was dialyzed overnight against 50 mM Tris-HCl (pH8.0). The dialysate was passed through a HiTrap DEAE FF 5 mL column equilibrated in 50 mM Tris-HCl (pH8.0) and concentrated by ultrafiltration. A further purification step was performed by size exclusion column chromatography with the superdex75 16/600 prep grade column (GE healthcare). The column was equilibrated in 20 mM Tris-HCl (pH8.0), 100 mM NaCl. The fractions containing FABP3 were pooled.

Delipidation by lipidex 1000 chromatography was performed to remove endogenous *E. coli* FAs from the purified FABP3. However, the procedure was unsuccessful because the X-ray crystal

structure analysis clearly exhibited an electron density consistent with some FAs bound to the active site. This technique did not seem to be favourable for delipidation of the FABP3 preparation. Consequently, apo-type FABP3 used in this study was prepared by refolding the purified protein at 310 K in 6 M guanidinium chloride to ensure >95% removal of intrinsic FAs that carried over from host *E. coli* cells. Specifically, the procedure for refolding the purified protein was performed according to the stepwise dialysis method reported by Tsumoto *et al.*[1,2] except that we did not add L-arginine and oxidized glutathione. The concentration of guanidinium chloride in the dialysis buffer (20 mM Tris-HCl at pH8.0 containing 5 mM dithiothreitol) was phased down (6, 4, 2, 1, 0.5, 0.25 and 0 M). The refolded proteins were concentrated to 0.1 mM with an Amicon ultra centrifugal filter unit (3000 NMWL, Merck Millipore, Bedford, MA, USA). The concentrated FABP3 was purified on a HiLoad 16/60 Superdex 75 prep-grade column (GE Healthcare, USA) equilibrated with 50 mM Tris-HCl (pH 8.0) containing 100 mM NaCl at 277 K.

Liposome preparation. Each FA analogue was incorporated into large unilamellar vesicles (LUVs) with a FA/phospholipid molar ratio at 1/10 and less, where DMPC was used as the matrix lipid because of its physical similarity to mammalian cell membrane. FA-containing LUVs were prepared by thin film method[3] followed by extrusion through polycarbonate membranes with a pore size of ϕ100 nm[4] Details of example procedure were shown below.

Fatty acids (FAs) were purchased from Sigma-Aldrich and Wako Pure Chemicals. 1,2-dimyristoyl-*sn*-glycero-3-phosphocholine (DMPC) was purchased from Avanti Polar Lipids as a dry powder. These chemicals were used without further purification. 3.7 mg of DMPC was dissolved in 2 mL of chloroform-methanol (1:1 vol:vol) with a FA of K^+ salt form at 1/11 (or 1/10) molar ratio in a glass vial. The organic solvent was removed under vacuum for more than 6 hours subsequent to evaporation with V-10 solvent evaporation system (Biotage, Uppsala, Sweden), the resultant lipid film was hydrated and suspended by using bath sonicator (ASU Cleaner, AS ONE, Osaka, Japan) into 500 μL of ITC buffer: 20 mM Tris-HCl, pH 8.0, and 100 mM NaCl (or 20 mM potassium phosphate, pH 7.0, and 100 mM NaCl). In case of unsaturated FAs, the vial was flushed with nitrogen gas after removal of organic solvent. The lipid suspension was then subjected to 3 cycles of a freeze-thaw treatment at –30 °C/40 °C to form multilamellar vesicles (MLVs). The resulting MLVs were extruded through a polycarbonate membrane with a 100 nm diameter pore size in LiposoFast extruder (Avestin Inc., Ottawa, Canada) to form LUVs. The FA concentration in the extruded LUV solution was determined by the 2-nitrophenylhydrazine (2-NPH) method[5] using a short- and long-chain fatty acid analysis kit (YMC, Kyoto, Japan).

Isothermal titration calorimetry. The ligand selectivity of FABP3 was systematically examined by isothermal titration calorimetry (ITC) with a liposome-mediated instillation of FA avoiding overestimation of the hydrophobic interaction. The ITC experiments were performed using a Nano ITC Low Volume system (*TA Instruments, New Castle, DE, USA*). Delipidated FABP3 was prepared as 0.2 mM solution in ITC buffer (20 mM Tris-HCl, pH 8.0, and 100 mM NaCl, or 20 mM potassium phosphate, pH 7.0, and 100 mM NaCl) by dilution subsequent to dialysis against the ITC buffer at 10~14 mg/mL of protein concentration. Apo FABP3 solution (210 μL of 0.2 mM) was titrated with 50 μL of 1 mM FA incorporated in 10 mM DMPC LUVs. In a typical experiment, 20-25 aliquots of 2-μl of FA-DMPC LUVs solution were injected to FABP3 solution with 3-min intervals at 37 °C. The number of active binding site per protein was observed to be between 0.3 and 0.9. In the other experiments for FA analogues (Table S1**b**) and some of SFAs, we used the phosphate only buffer (20 mM K-phosphate pH7.0 and 100 mM NaCl) at 310 K, where the number of active binding site per protein was between 0.6 and 0.9. Since the crystals of FABP3 were prepared with the Tris buffer system, we mainly adopted the thermodynamic parameters obtained under the Tris buffer conditions. Each experiment was accompanied by a ligand dilution in which the FA-DMPC LUV solution was titrated against the ITC buffer. The ligand dilution data were subtracted from the corresponding binding data before analyses. Obtained ITC data were fitted using the NanoAnalyze software (TA instruments) based on the independent model (single binding site) to deduce thermodynamic parameters. All experiments were repeated a minimum of three times. Mean and standard error values were calculated from the three sets of data.

X-ray co-crystallography. On the purpose of obtaining clearer electron density maps for FA alkyl chains, the delipidated FABP3 at a concentration of 2 mg/ml were incubated for 1 h at 310 K with an excessive amount (>1.5-fold molar) of FA. The FABP3 in complex with FAs were concentrated to approximately 20 mg/ml for crystallization. The crystals were obtained using the sitting drop vapour diffusion method. The co-crystals of the FABP3 were each grown in 1.0 μL of protein solution and 1 μL of reservoir solution (100 mM Tris-HCl, 55 % PEG400, pH 8.0−8.5) equilibrated against 1.0 mL of the reservoir solution at 293 K. These crystals were directly flash-cooled in a stream of cold nitrogen gas at 100 K with reservoir solution as a cryoprotectant. X-ray diffraction data were collected by beamlines BL38B1 and BL44XU from SPring-8 synchrotron radiation sources (Harima, Japan). Diffraction data were processed using the *HKL2000* program.[6]

The deposited FABP3 structure coordinate (PDB ID 1HMT) was used as starting models for the structural analysis. Structural refinements were carried out using a restrained least-squares refinement method in *Refmac* software[7] as implemented within the *CCP4* package. Anisotropic refinements were carried out using the program *SHELXL*. [8] The geometry of the refined model was validated by the program *PROCHECK*.[9] The data collection and refinement statistics are

summarized in Table S1. The final atomic coordinates and structure-factor amplitudes (PDB entries 4TJZ, 4TKB, 4TKH, 4TKJ, and 3WVM) have been deposited in the Worldwide Protein Data Bank (wwPDB; http://www.wwpdb.org) and the Protein Data Bank Japan at the Institute for Protein Research, Osaka University, Suita, Osaka, Japan (PDBj; http://www.pdbj.org/).

WaterMap calculation. The protocols involved in the WaterMap calculations are described in previous works.[10] Input protein structures were prepared using the Protein Preparation Wizard in the Maestro (version 9.8)[11] molecular modelling suite. Amino acid residues outside of a 20 Å shell around SFAs were removed and the system was solvated in a TIP4P[12] water box extending at least 10.0 Å beyond the truncated protein in all directions. An 8.0 ns MD simulation was performed following a standard WaterMap relaxation protocol with 5.0 kcal/mol positional constraints, except for Lys58 and Asp77, to facilitate equilibration of the water molecules in the binding site. Water molecules from the frames saved at 1.5 ps intervals in the simulation were clustered into distinct hydration sites, and the excess entropy and enthalpy were calculated relative to bulk solvent according to the inhomogeneous solvation theory.[13]

Molecular dynamics simulation. All simulations of the FABP3-FA complexes were performed at 310 K with the program 'MARBLE'.[14] The CHARMM36/CMAP for the protein,[15] CHARMM36[16] for FA and TIP3P for water[17] were used as the force-field parameters. Periodic boundary conditions and the particle mesh Ewald method were applied. The Lennard-Jones potential was smoothly switched to zero over the range 8 – 10 Å. The symplectic integrator for rigid bodies was used and CH_x, NH_x (x = 1, 2, 3), SH and OH groups were treated as rigid bodies. The time step was set to 2.0 fs.

For FA = C10:0 to C16:0, the initial coordinates of our simulations were taken from the final stages of the crystal structure refinements described in the previous section. For FA = C18:0, the crystal structure with a resolution of 1. 37 Å at a later stage of the refinement by our group was used as the initial structure. Polyethylene glycol and glucosamine phosphate molecules in the crystal structures were removed, and the oxygen atoms of these molecules were replaced by water oxygen atoms except for the phosphate oxygen atoms. The first methionine residue of FABP3 was removed. Using PROPKA3.1,[18] we estimated that all protein residues adopt the default protonation states and the FAs are in a deprotonated state. The protein, bound FA and crystal water molecules were placed in a cubic box to accommodate a minimum water shell thickness of 12 or 13.5 Å. Two Na^+ ions were added in bulk solution to neutralize the system. The resulting systems contain over 31,500 atoms. Before starting the MD simulations, the systems were minimized to remove unfavourable contacts.

For the equilibration, the systems were gradually heated to 310 K for 100 ps under the *NPT* condition at 1 atm with the constraints on the positions of all non-hydrogen atoms of the protein and

bound FA. The same constraints were kept for the next 100 ps, and then gradually removed over a period of 150 ps. Subsequent 650 ps runs with no positional constraints were discarded as the further equilibration. After the equilibration runs, 20 ns production run were performed under the *NPT* condition at 1 atm and 310 K for all systems. The snapshots were saved every 1 ps.

During the 20 ns trajectories, the protein backbone and FAs fluctuated around the crystal structures. The protein backbone and the FA atoms in every snapshot were superimposed as one unit on the corresponding energy-minimized structure using the method proposed by Kabsch.[19,20] The root-mean-squared fluctuations of FAs in Figure S4c were calculated using the structures after the superimposition.

Structure Comparison between protein molecules in same protein families.

We obtained the lists of PDB and chain IDs belonging to the same protein family from Pfam database (version 27.0).[21,22] Because the database was not updated for nearly one year as of February 2014, we added some crystal structures newly-deposited in the PDB to the lists referring to the sequence neighbor lists in PDBj.[23]

Residue numbers are sometimes different between the different PDB files of the same protein, therefore equivalent residues in two molecules were identified through the sequence alignment with 'Clustal omega'[24] Then one of the two protein molecules was superimposed on the other molecule with the method proposed by Kabsch.[19,20] For the superimposition, Cα atoms of all residues were used, but the ones of the residues missing in at least one molecule were not used in the calculation. Finally, root-mean-squared difference (RMSD) for the Cα atoms between the two molecules was calculated. The pair which shows the largest RMSD value among the all protein molecule pairs in the protein family was tabulated in Table S5.

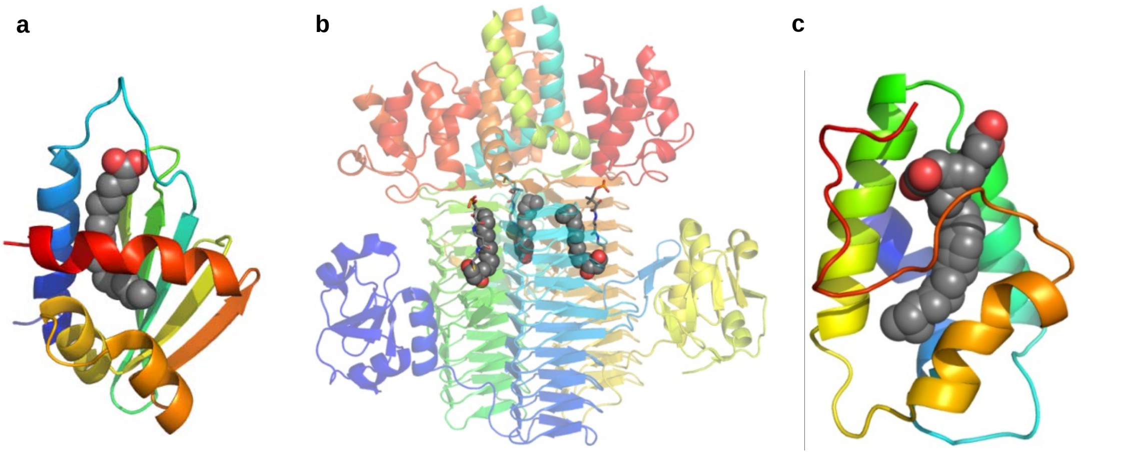

Figire S1. **FA structures in co-crystals with proteins.** (**a**) C16:0/yellow fever mosquito sterol carrier protein-2 like-3 (1PZ4). (**b**) 3-hydroxy C14:0-CoA/*E. coli* acyl carrier protein-UDP-3-O-(3-hydroxymyristoyl)glucosamine N-acyltransferase (4IHF), (**c**) C18:1 n-6c/maize nonspecific lipid-transfer protein showing a double conformation for FA molecule (1FK5,). FA alkyl chains are shown in spheres.

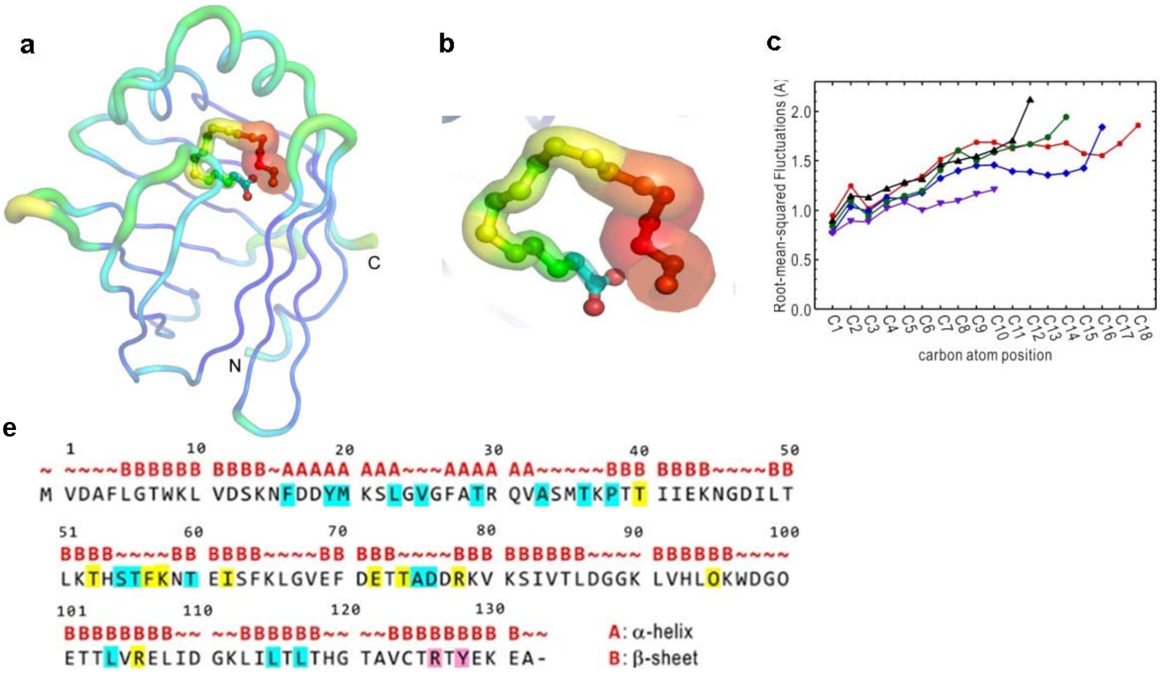

Figure S2. **Thermal fluctuation of FABP3-FA complex.** (**a**) and (**b**) Fluctuations in the FABP3-C18:0 co-crystal structure obtained at room temperature shown with temperature factor (smaller in thinner and blue, and larger in thicker and red). (**c**) Fluctuations of five SFAs bound to FABP3 in solution state deduced from 20 ns of MD simulations (C10:0 in violet, C12:0 in black, C14:0 in green, C16:0 in blue, and C18:0 in red). (**e**) Amino acid sequence of FABP3; Blue highlight: residues in contact with the FA; yellow: residues in contact with water in the pocket; pink: residues of hydrogen-bonded to the carboxylate of the FA.

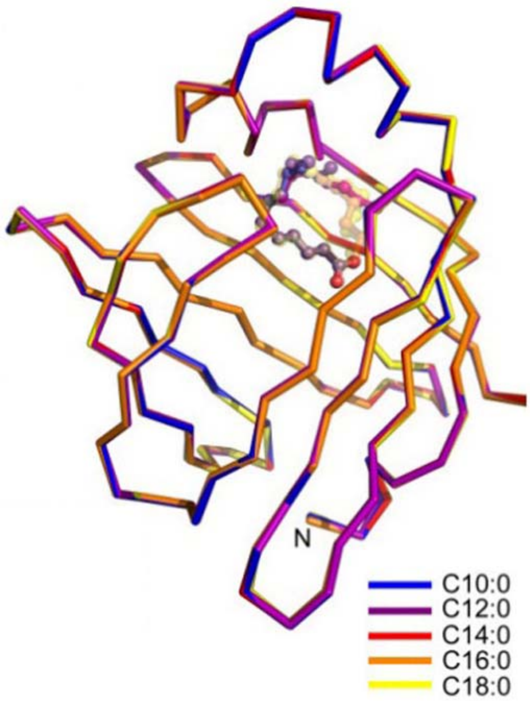

Figure S3. **FABP3-SFA binding structures at the cryogenic temperature.** Conformations of FABP3 main chain superposed for five SFA-co-crystals. The root-mean-square deviation (RMSD) was 0.13Å

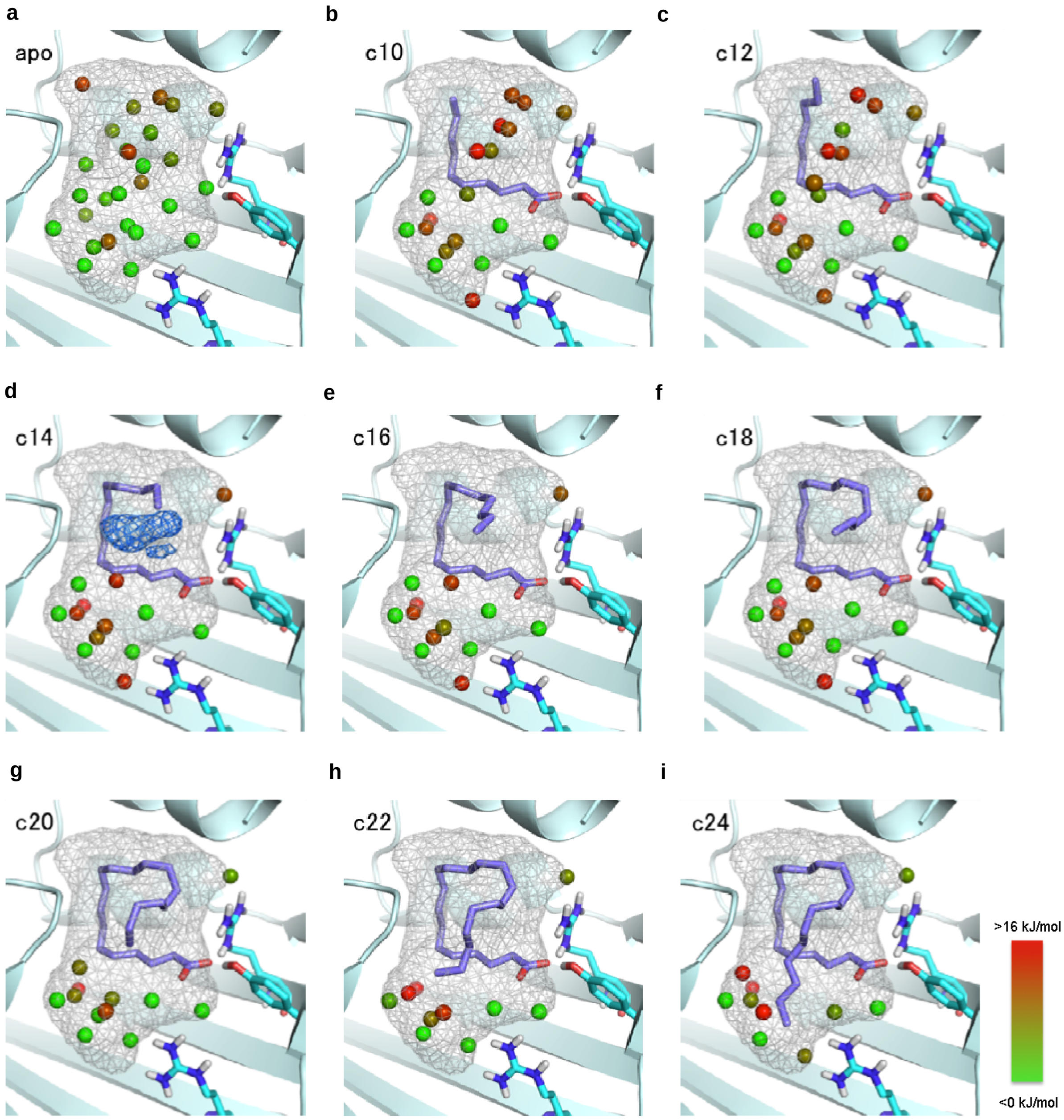

***Figure S4*. Hydration sites identified by WaterMap in the binding site of FABP3 with SFAs.** WatarMap analysis results for FABP3 complexed with C10:0 – C24:0 (**b** – **i**) and its apo form (**a**). The structure analysis of the apo form **a** obtained by deleting the ligand from the C18:0/FABP3 complex identified 18 water molecules (27 hydration sites) within the binding site (see also Figure 5**a**). For FABP3 complexed with C20:0 – C24:0, whose X-ray structures are not available, the binding poses of SFAs are computationally modeled based on the binding pose of C18:0. Colour gradation of hydration sites is based on the free energy relative to bulk water with stable sites shown in green and unstable sites in red. In the binding site of the complex bound with

C14:0, a dewetted cavity (surface area = 32.3 Å^2, shown in blue mesh), a region where virtually no water density was found from the MD trajectory, was found. The terminal parts of the extended chains of C20:0 – C24:0 are predicted to intrude into the region occupied by Cluster 1 and disrupt the hydrogen bond network resulted in the formation of high energy hydration sites.

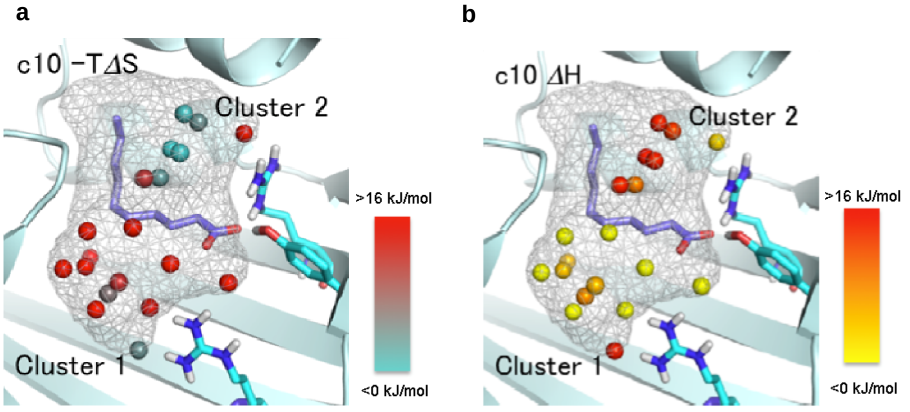

***Figure S5*. Thermodynamic signature of the water molecules in Clusters 1 and 2 calculated by WaterMap.** *ΔH* and –*TΔS* (relative to bulk solvent) of the hydration sites found in the binding site of FABP3 incorporating C10:0 are shown. The differences in their thermodynamic profiles for Clusters 1 and 2 are clearly seen. **a**) Entropy (–*TΔS*) of the hydration sites. **b**) Enthalpy of the hydration sites. Large –*TΔS* values of Cluster 1 mean translational and rotational freedom of the water molecules are heavily restricted. On the other hand, enthalpies of Cluster 1 water molecules are mostly similar to the value of bulk water. These observations strongly suggest the presence of a strong hydrogen bond network in Cluster 1. In contrast, the thermodynamic signature of Cluster 2 water molecules suggest that they are loosely hydrogen bonded to each other and have larger freedom of movement. The preferential displacement of Cluster 2 water molecules by the extended chain of SFAs is well explained by this difference in the thermodynamic profiles.

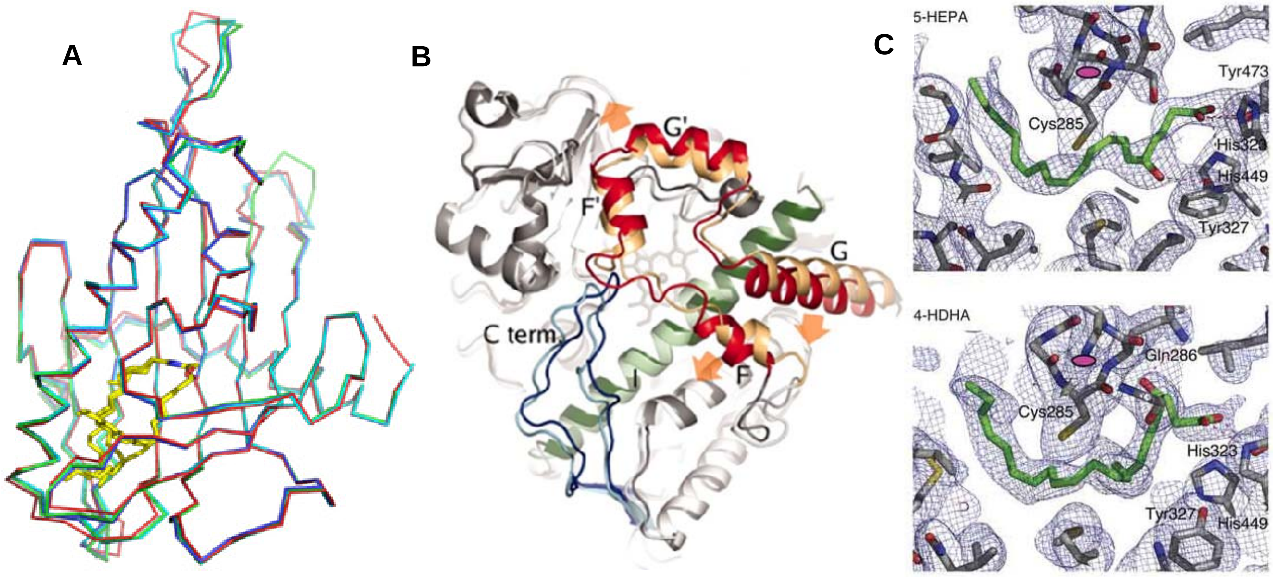

Figure S6. **Examples of promiscuous recognition of lipids and hydrophobic ligands by rigid protein (A) and flexible proteins (B and C).**

Among other proteins, the above three lipid-binding proteins are focused; CERT is the relatively rigid proteins that bind various ceramides,[25] and cytochrome P450[26,27] and peroxisome proliferator-activated receptor (PPAR)[28-30] are known to recognize their ligands in an induced-fit manner. See ***Table S5*** for detailed data.

(**A**) CERT: Main chains of the apo- and C_6-, C_{16}-, and C_{18}-ceramide bound structures of the CERT START domain, particularly near the binding cavity, are largely superimposed. Red, apo-form; green, blue, and cyan, C_6-, C_{16}-, and C_{18}-ceramide bound forms, respectively.[25] The image was taken from reference[25] © The National Academy of Sciences of the United States of America.

(**B**) Cytochrome P450 3A4: A superposition of the ketoconazole (hydrophobic ligand, not shown) complex (2J0C, dark colours) and the ligand-free structures (1TQN, light colours). Note that the structures of α-helices F/F', G/G' and a C-terminus loop undergo large conformation changes by ligand binding as depicted by orange arrows. The images were taken from reference.[26] © The National Academy of Sciences of the United States of America.

(**C**) PPARγ: The structures of ligand-binding cavities of PPARγ with 5-hydroxyeicosapentaenoic acid (5-HEPA) and 4-hydroxydocosahexaenoic acid (4-HDHA). Note the differences in the conformation and orientation of the ligands and surrounding amino acid residues. The image was taken from reference[30] with permission. © Nature Publishing Group.

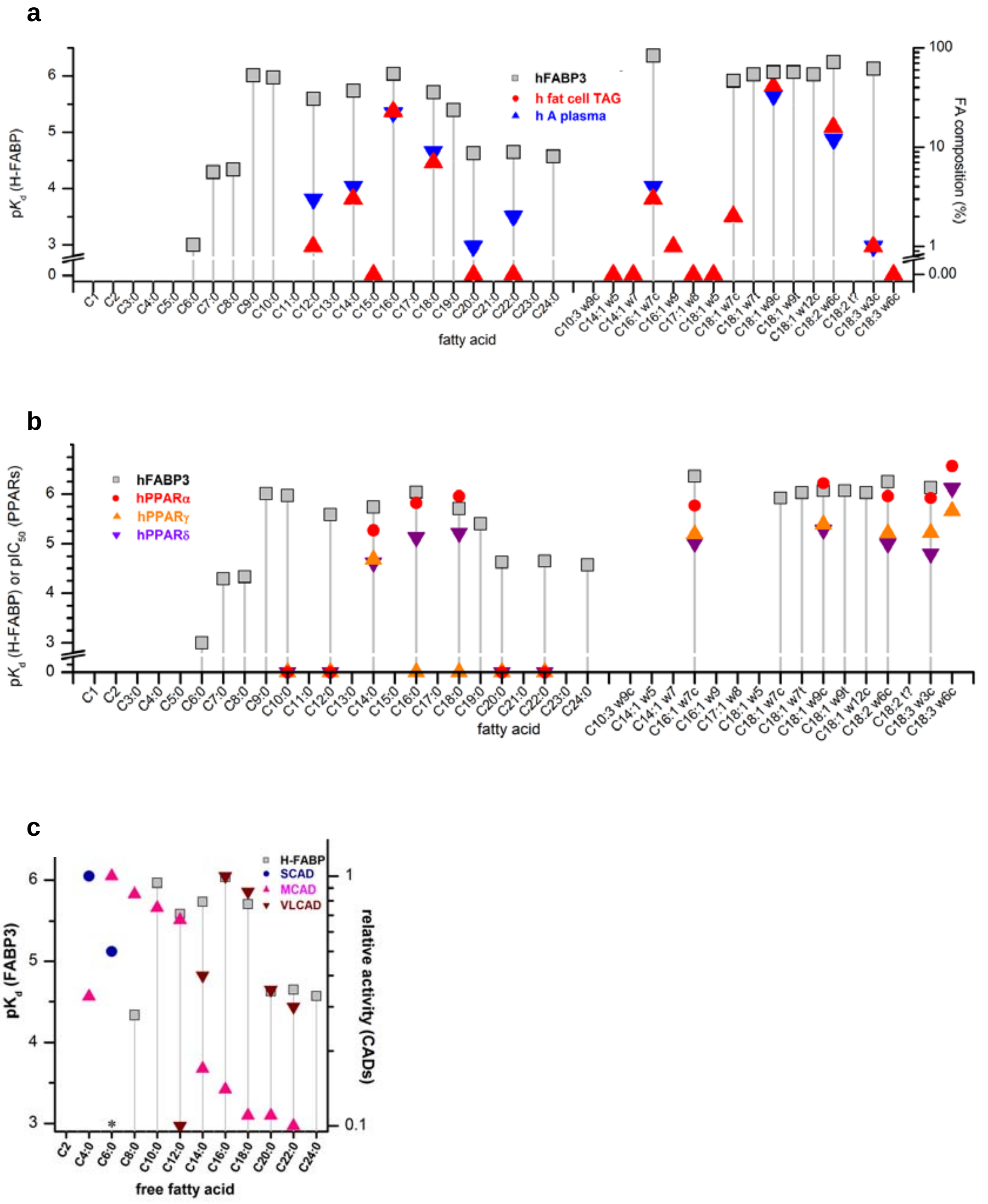

***Figure S7*. Overlay plots of FA selectivity profiles.** (**a**) FA compositions in human fat cell tryacylglycerol (red circles)[31] and artery plasma (blue triangles)[32] in percentage (right axis) and FABP3 FA affinities in pK_d (gray squares, left axis). (**b**) FA binding affinities to three subtypes of human peroxisome proliferator-activated receptors (PPARs α in red circle, γ in orange triangles, and δ in violet inverted triangles)[33] and FABP3 (gray squares). FA affinities to PPARs are shown in pIC_{50} evaluated by inhibition assay against ^{3}H-labeled ligands.[33] (**c**) Overlay plot of SFA selectivity profiles for FABP3 (gray squares) and acyl-CoA dehydrogenases short-chain (SCAD, blue circles), medium-chain (MCAD, magenta triangles) and long-chain (VLCAD, dark red inverted triangles)

involved in the first reaction in mitochondrial β-oxidation.[34] *The affinity of C6:0 FA was below the detection limit. Water-soluble short SFAs of C6-C8 and the FAs of C10-C18 are also favourable substrates of the mitochondrial β-oxidation cascade.[34] The overlapping chain-length preference between FABP3 and acyl-CoA dehydrogenases indicates that FABP3 selectively binds water-insoluble "fuel" FAs as the substrates for mitochondrial energy production.

***Table S1.* FABP3 binding parameters obtained from liposomal ITC experiments.**

a: FABP3 affinity and thermodynamic parameters for FAs*

fatty acid	K_d (M)			ΔH (kJ/mol)			$-T\Delta S$ (kJ/mol)		
*SFA***									
C6:0	n.o.***			–			–		
C7:0	5.08E-05			-62.04			36.52		
C8:0	4.61E-05	±	3.33E-05	-79.46	±	7.93	52.22	±	6.38
C9:0	9.69E-07			-79.27			43.55		
C10:0	1.07E-06	±	3.64E-07	-69.14	±	5.40	33.38	±	6.11
C12:0	2.56E-06	±	6.97E-07	-81.35	±	5.81	47.93	±	6.22
C14:0	1.84E-06	±	7.64E-07	-79.30	±	7.38	44.30	±	9.12
C16:0	9.13E-07	±	8.45E-08	-98.55	±	22.98	62.63	±	23.11
C18:0	1.95E-06	±	6.12E-07	-96.63	±	6.47	62.44	±	6.38
C19:0	3.97E-06			-75.81			43.71		
C20:0	2.37E-05	±	5.40E-06	-91.89	±	23.37	64.24	±	23.12
C22:0	2.25E-05	±	7.61E-06	-30.71	±	11.94	2.64	±	12.64
C24:0	2.70E-05	±	8.84E-07	-8.91	±	1.01	-18.21	±	0.94
UFA									
C16:1 n-7c	4.39E-07	±	1.62E-07	-137.91	±	27.96	99.81	±	28.65
C18:1 n-7c	1.21E-06	±	3.14E-07	-80.77	±	7.31	45.40	±	7.59
C18:1 n-7t	9.32E-07	±	6.41E-08	-113.25	±	10.89	77.39	±	10.70
C18:1 n-9c	8.48E-07	±	4.20E-08	-83.68	±	5.68	47.59	±	5.56
C18:1 n-9t	8.46E-07	±	2.10E-07	-87.52	±	7.53	51.23	±	7.01
C18:1 n-12c	9.35E-07	±	1.56E-07	-82.10	±	4.02	45.21	±	2.68
C18:2 n-6c	5.58E-07	±	2.54E-07	-104.39	±	9.12	66.71	±	10.01
C18:3 n-3c	7.36E-07	±	2.15E-07	-103.54	±	11.15	66.87	±	10.60

*evaluated by liposomal ITC in 20 mM Tris-HCl pH8.0 and 100 mM NaCl at 310 K. Experiments were performed triplicate except for C7:0, C9:0 and C19:0.**For saturated FAs (C6:0-C20:0), another series of ITC measurements was carried out under the phosphate buffer conditions at 310 K, which provided similar thermodynamic parameters. ***not observed.

b: FABP3 affinity and thermodynamic parameters for FA analogues*

ligand	K_d (M)			ΔH (kJ/mol)			$-T\Delta S$ (kJ/mol)		
C10:0	1.63E-06	±	2.63E-07	-80.66	±	4.02	46.19	±	4.47
C16:0	8.85E-07	±	4.29E-08	-44.86	±	0.10	9.51	±	0.83
retinoic acid	n.o.**			–			–		
prostaglandin E2	5.45E-05	±	2.62E-05	-66.81	±	14.80	41.32	±	16.09
phytanic acid	>5.00E-04			–			–		
jasmonic acid	2.45E-05	±	1.98E-07	-34.81	±	0.46	7.65	±	0.44

*evaluated by liposomal ITC in 20 mM K-phosphate pH7.0 and 100 mM NaCl at 310 K. ** not observed.

Table S2. **X-ray data processing and structure determination statistics for FABP3 in complex with the fatty acids.**

FA complexes	C10:0 complex	C12:0 complex	C14:0 complex	C16:0 complex	C18:0 complex
Source	SPring-8 BL44XU	SPring-8 BL44XU	SPring-8 BL44XU	SPring-8 BL44XU	SPring-8 BL44XU
Wavelength (Å)	0.80	0.80	0.80	0.80	0.80
Temperature (K)	100	100	100	100	100
Space group	$P2_12_12_1$	$P2_12_12_1$	$P2_12_12_1$	$P2_12_12_1$	$P2_12_12_1$
Unit-cell parameters (Å)					
a, b, c	54.5, 69.7, 33.9	54.4, 69.7, 33.8	54.6, 69.5, 33.8	54.7, 69.9, 33.7	54.6, 69.4, 33.8
Resolution (Å)	50 – 0.87	50 – 0.86	50 – 0.93	50 – 0.87	50 – 0.88
(High-resolution shell)	(0.89 – 0.87)	(0.87 – 0.86)	(0.95 – 0.93)	(0.89 – 0.87)	(0.90 – 0.88)
No. of reflections	1,061,542	1,298,725	997,728	1,021,456	1,249,690
Oscillation angle (°)	0.5	0.5	0.5	0.5	0.5
Total rotation angle (°)	360	360	360	360	360
No. of unique reflections	104,970	107,896	86,123	106,214	100,773
Redundancy	10.1 (8.0)	12.0 (5.4)	11.6 (8.9)	9.6 (7.5)	12.4 (6.6)
$\langle I/\sigma(I) \rangle$	10.1 (3.4)	9.8 (2.3)	10.1(3.5)	10.3 (3.7)	8.6 (2.5)
Completeness (%)	98.6 (97.5)	98.0 (82.0)	98.9 (97.9)	99.5 (99.9)	98.6 (87.0)
R_{merge} (%)[a]	7.1 (40.4)	6.6 (39.2)	7.9 (39.9)	6.8 (41.8)	6.6 (42.3)
Mosaicity (°)	0.49	0.27	0.38	0.51	0.25
Refinement					
Resolution range (Å)	42.94 – 0.87	42.94 – 0.86	42.91 – 0.93	43.08 – 0.87	42.89 – 0.88
No. of reflections	99,487	102,442	81,538	100,656	95,629
Data cutoff [Sigma(Fo)]	none	none	none	none	none
R_{cryst}[b] / R_{free}[c]	0.10 / 0.11	0.10 / 0.12	0.11 / 0.12	0.10 / 0.12	0.10 / 0.11
No. of water molecules	193	194	165	189	174
No. of fatty acids	1	1	1	1	1
RMSD bond length (Å)	0023	0023	0.022	0.025	0.023
RMSD bond angle (°)	2.3	2.6	2.3	2.5	2.3
PDB ID	4TJZ	4TKB	4TKH	4TKJ	3WVM

[a]$R_{merge} = \sum_{hkl}\sum_i |I_i(hkl) - \langle I(hkl)\rangle| / \sum_{hkl}\sum_i I_i(hkl)$, where $I_i(hkl)$ is the *i*th observed intensity of reflection *hkl* and $\langle I(hkl)\rangle$ is the average intensity over symmetry-equivalent measurements. Values in parentheses are for the highest resolution shell.

[b]$R_{cryst} = \sum||F_o| - |F_c||/\sum|F_o|$ calculated from 95% of the data, which were used during the course of the refinement.

[c]$R_{free} = \sum||F_o| - |F_c||/\sum|F_o|$ calculated from 5% of the data, which were used during the course of the refinement.

***Table S3.* Properties of hydration sites in clusters 1 and 2 in C10-FABP3 complex.**

Site[a]	Cluster	Occupancy[b]	ΔH^c (kJ/mol)	$-T\Delta S^c$ (kJ/mol)	ΔG^c (kJ/mol)	#HB(WB)[d]	#HB(PW)[e]
1	2	0.96	-0.54	15.22	14.68	0.79	2.06
2	2	0.86	12.90	11.99	24.89	0.91	1.68
3	2	0.69	11.45	6.89	18.33	1.87	0.00
4	2	0.57	6.68	6.39	13.07	2.41	0.00
5	2	0.38	13.11	3.82	16.92	1.68	0.32
6	2	0.37	13.69	4.40	18.09	1.21	0.72
7	2	0.35	20.70	3.40	24.10	1.81	0.00
8	1	1.00	-50.23	22.52	-27.71	0.83	1.92
9	1	1.00	-16.30	19.21	0.29	2.27	0.98
10	1	1.00	2.53	19.74	22.27	0.43	2.67
11	1	0.99	-36.30	20.57	-15.72	1.72	0.97
12	1	0.98	-39.70	19.83	-19.87	1.21	1.99
13	1	0.98	-28.54	18.58	-9.96	1.92	1.01
14	1	0.98	1.53	16.22	17.75	1.64	0.29
15	1	0.95	-4.11	15.76	11.66	2.15	0.67
17	1	0.92	-13.73	14.60	0.87	1.77	0.81
18	1	0.91	2.24	12.65	14.89	0.78	1.76
19	1	0.66	5.27	8.67	13.94	1.50	0.02
20	1	0.48	14.48	6.89	21.36	0.39	0.90

[a]The site numbers are shown in the figure below. [b]Occupancy is the fraction of the MD frames in which a water molecule is found at the hydration site, in the total number of frames recorded. [c]ΔH, ΔS and ΔG of the hydration siteswere calculated based on the free energy relative to bulk water. [d]#HB(WB) is the number of the hydrogen bonds between the water molecules. [e]#HB(PW) is the number of the hydrogen bonds between the water molecules and the protein.

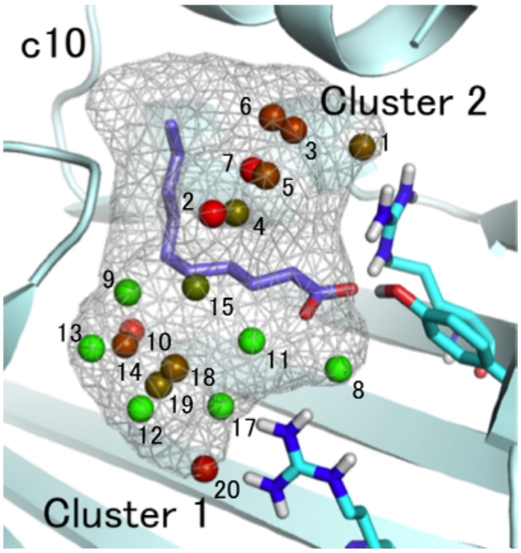

Hydration site numbers are indicated in C10-FABP3 complex. Colour gradation is based on the free energy relative to bulk water in the same way as Figure S4.

Table S4. **The sums of the thermodynamic parameters of the water molecules in Cluster 1 calculated by WaterMap*.**

Ligand	ΔH (kJ/mol)	$-T\Delta S$ (kJ/mol)	ΔG (kJ/mol)	Sum Occupancy
C10:0	-171.64	191.51	19.87	10.83
C12:0	-171.77	202.34	30.57	11.01
C14:0	-159.37	193.38	34.01	10.86
C16:0	-181.31	201.26	19.91	10.67
C18:0	-178.12	202.01	23.85	10.56

*Note that the parameters do not vary significantly for the FABP3 proteins complexed with various length of saturated fatty acid chains (C10:0–18:0). These data imply that the interactions between the extended part of the fatty acids and Cluster 1 water molecule network are insignificant.
ΔH, ΔS and ΔG of the hydration sites were calculated based on the free energy relative to bulk water.

Table S5. **Largest RMSD values in the protein families of START, PPARs and CYP450, and the pairs of protein molecules**

UniProt entry name	Protein name	**PDB accession codes_chain IDs** and structure titles deposited in PDB		RMSD
		Reference Structure	Moving Structure	All Cα atoms (Å)
C43BP_ HUMAN	CERT START	**2E3O_A** (Crystal structure of CERT START domain in complex with C6-ceramide (P212121))[25]	**2E3R_A** (Crystal structure of CERT START domain in complex with C18-ceramide (P1)) [25]	1.323
CP2B4_ RABIT	rabbit Cytochrome P450 2B4	**2BDM_A** (Structure of Cytochrome P450 2B4 with Bound Bifonazole)[35]	**3TK3_B** (Cytochrome P450 2B4 mutant L437A in complex with 4-(4-chlorophenyl)imidazole)[36]	5.934
CP3A4_ HUMAN	human Cytochrome P450 3A4	**2J0D_B** (Crystal structure of human P450 3A4 in complex with erythromycin)[26]	**4K9X_A** (Complex of human CYP3A4 with a desoxyritonavir analog)[37]	2.658
CPXA_ PSEPU	Camphor 5-mono-oxygenase	**3FWF_A** (Ferric camphor bound cytochrome P450cam containing a Selenocysteine as the 5th heme ligand, monoclinic crystal form)[38]	**4JWS_B** (Crystal structure of Cytochrome P450cam-putidaredoxin complex)[39]	2.650
CPXB_ BACME	cytochrome P450 102	**1SMI_A** (A single mutation of P450 BM3 induces the conformational rearrangement seen upon substrate-binding in wild-type enzyme)[40]	**4KF0_B** (Structure of the A82F P450 BM3 heme domain)[41]	2.819
PPARA_ HUMAN	human PPAR-alpha	**1KKQ_A** (Crystal structure of the human PPAR-alpha ligand-binding domain in complex with an antagonist GW6471 and a SMRT corepressor motif)[42]	**3G9I_A** (Aleglitazar, a new, potent, and balanced PPAR alpha/gamma agonist for the treatment of type II diabetes)[43]	4.560
PPARG _HUMA N	human PPAR-ga mma	**2HWQ_B** (Structural basis for the structure-activity relationships of Peroxisome Proliferator-Activated Receptor agonists)[44]	**2Q4Y_B** (Ensemble refinement of the protein crystal structure of At1g77540-coenzyme A complex)[45]	3.788
PPARD_ HUMAN	human PPAR- delta	**2J14_A** (3,4,5-trisubstituted isoxazoles as novel PPARdelta agonists: PART2)[46]	**2Q5G_A** (Ligand binding domain of PPAR delta receptor in complex with a partial agonist)[47]	3.510

References

1. K. Tsumoto, K. Shinoki, H. Kondo, M. Uchikawa, T. Juji, I. Kumagai, *J. Immunol. Methods* **2008,** *219,* 119–129.
2. K. Tsumoto, M. Umetsu, H. Yamada, T. Ito, S. Misawa, I. Kumagai, *Protein Eng.* **2003,** *16,* 535–541.
3. A. D. Bangham, M. M. Standish, J. C. Watkins, *J. Mol. Biol.* **1965,** *13,* 238–252.
4. R. C. MacDonald, R. I. MacDonald, B. Ph. M. Menco, K. Takeshita, N. K. Subbarao, L. Hu, *Biochim. Biophys. Acta* **1991,** *1061,* 297–303.
5. H. Miwa, C. Hiyama, M. Yamamoto, *J. Chromatogr.* **1985,** *321,* 165–174.
6. Z. Otwinowski, W. Minor, *Methods Enzymol.* **1997,** *276,* 307–326.
7. A. T. Brünger, P. D. Adams, G. M. Clore, W. L. DeLano, P. Gros, R. W. Grosse-Kunstleve, J.-S. Jiang, J. Kuszewski, M. Nilges, N. S. Pannu, R. J. Read, L. M. Rice, T. Simonson, G. L. Warren, *Acta Cryst.***1998,** *D54,* 905–921.
8. G. M. Sheldrick, T. R. Schneider, Methods. Enzymol. 1997, 277, 319-343.
9. R. A. Laskowski, M. W. MacArthur, D. S. Moss, J. M. Thornton, *J. Appl. Cryst.* **1993,** *26,* 283–291.
10. T. Beuming, Y. Che, R. Abel, B. Kim, V. Shanmugasundaram, W. Sherman, *Proteins* **2012,** *80,* 871–883.
11. S. G. Madhavi, M. Adzhigirey, T. Day, R. Annabhimoju, W. J. Sherman, *Comput. Aided Mol. Des.* **2013,** *27,* 221–234.
12. W. L, Jorgensen, J. D. Madura, *Mol. Phys.* **1985,** *56,* 1381–1392.
13. T. Lazaridis, *J. Phys. Chem. B* **1998,** *102,* 3531–3541.
14. M. Ikeguchi, *J. Comput. Chem.* **2004,** *25,* 259–541.
15. R. B. Best, X. Zhu, J. Shim, P. E. M. Lopes, J. Mittal, M. Feig, A. D. MacKerell, Jr. *J. Chem. Theory Comput.***2012, 8,** 3257–3273.
16. J. B. Klauda, R. M. Venable, J. A. Freites, J. W. O'Connor, D. J. Tobias, C. Mondragon-Ramirez, I. Vorobyov, A. D. MacKerell, Jr., R. W. Pastor, *J. Phys. Chem. B*, **2010,** *114,* 7830–7843.
17. W. L. Jorgensen, J. Chandrasekhar, J. D. Madura, R. W. Impey, M. L. Klein, M. L. *J. Chem. Phys.* **1983,** *79,* 926–935.
18. C. R. Søndergaard, M. H. M. Olsson, M. Rostkowski, J. H. Jensen, *J. Chem. Theory Comput.* **2011,** *7,* 2284–2295.
19. W. Kabsch, *Acta Cryst., A* **1972,** *32,* 922–923.
20. W. Kabsch, *Acta Cryst., A*, **1974,** 34, 827–828.
21. R. D. Finn, A. Bateman, J. Clements, P. Coggill, R. Y. Eberhardt, S. R. Eddy, A. Heger, K. Hetherington, L. Holm, J. Mistry, E. L. L. Sonnhammer, J. Tate, M. Punta, *Nucleic Acids*

*Research,***2014,** *42,* D222-D230.

22. http://pfam.xfam.org/
23. A. R. Kinjo, H. Suzuki, R. Yamashita, Y. Ikegawa, T. Kudou, R. Igarashi, Y. Kengaku, H. Cho, D. M. Standley, A. Nakagawa, H. Nakamura, H. *Nucleic Acids Research*, **2012,** *40,* D453-460.
24. F. Sievers, A. Wilm, D. Dineen, T. J. Gibson, K. Karplus, W. Li, R. Lopez, H. McWilliam, M. Remmert, J. Söding, J. D. Thompson, D. G. Higgins, *Mol. Sys. Biol.* **2011,** *7,* 539.
25. N. Kudo, K. Kumagai, N. Tomishige, T. Yamaji, S. Wakatsuki, M. Nishijima, K. Hanada, R. Kato, *Proc. Natl. Acad. Sci USA*. 2008, *105*, 488–493.
26. M. Ekroos, T. Sjögren, *Proc. Natl. Acad. Sci USA*. 2006, 103, 13682–13687.
27. D. C. Haines, B. Chen, D. R. Tomchick, M. Bondlela, A. Hegde, M. Machius, J. A. Peterson, *Biochemistry,* **2008,** *47,* 3662-3670.
28. V. Zoete, A. Grosdidier, O. Michielin, *Biochim. Biophys. Acta*, 2007, *1771*, 915–925.
29. G. Pochetti, C. Godio, N. Mitro, D. Caruso, A. Galmozzi, S. Scurati, F. Loiodice, G. Fracchiolla, P. Tortorella, A. Laghezza, A. Lavecchia, E. Novellino, F. Mazza, M. Crestani, *J. Biol. Chem.* **2007,** *282,* 17314-17324.
30. T. Itoh, L. Fairall, K. Amin, Y. Inaba, A. Szanto, B. L. Balint, L. Nagy, K. Yamamoto, J.W.R. Schwabe, *Nat Struct Mol Biol.* **2008,** *15*, 924–931.
31. T. Raclot, D. Langin, M. Lafontan, R. Groscolas, *Biochem. J.* **1997,** *324,* 911–915.
32. K. Halliwell, B. A. Fielding, J. S. Samra, S. M. Humphreys, K. N. Frayn, *J. Lipid Res.* **1996,** *37,* 1842–1848.
33. H. E. Xu, M. H. Lambert, V. G. Montana, D. J. Parks, S. G. Blanchard, P. J. Brown, D. D. Sternbach, J. M. Lehmann, G. B. Wisely, T. M. Willson, S. A. Kliewer, M. V. Milburn, *Mol. Cell.* **1999,** *3,* 397–403.
34. R. J. Wanders, J. P. Ruiter, L. IJlst, H. R. Waterham, S. M. Houten, S. M. *J. Inherit. Metab. Dis.* **2010,** *33,* 479–494.
35. Y. Zhao, M. A.White, B. K. Muralidhara, L. Sun, J. R. Halpert, C. D. Stout, *J. Biol. Chem.***2006,** *281,* 5973-5981.
36. P. R. Wilderman, S. C. Gay, H. H. Jang, Q. Zhang, C. D. Stout, J. R. Halpert, *FEBS J.*, **2012,** *279,*1607-1620.
37. I. F. Sevrioukova, T. L. Poulos, *Biochemistry*, **2013,** *52,* 4474-4481.
38. C. Aldag, I. A. Gromov, I. Garcia-Rubio, K. von Koenig, I. Schlichting, B. Jaun, D. Hilvert, *Proc. Natl. Acad. Sci. USA*, **2009,** *106,* 5481-5486.
39. S. Tripathi, H. Li, T. L. Poulos, *Science*, **2013,** *340,* 1227-1230.
40. M. G. Joyce, H. M. Girvan, A. W. Munro, D. Leys, *J. Biol. Chem.* **2004,** *279,* 23287-23293.
41. C. F. Butler, C. Peet, A. E. Mason, M. W. Voice, D. Leys, A. Munro, *J. Biol. Chem.***2013,** *288,* 25387-25399.

42. H. E. Xu, T. B. Stanley, V. G. Montana, M. H. Lambert, B. G. Shearer, J. E. Cobb, D. D> McKee, C. M. Galardi, K. D. Plunket, R. T. Nolte, D. J. Parks, J. T. Moore, S. A. Kliewer, T. M. Willson, J. B. Stimmel, *Nature* **2002,** *415,* 813-817.
43. A. Benardeau, J. Benz, A. Binggeli, D. Blum, M. Boehringer, U. Grether, H. Hilpert, B. Kuhn, H. P. Marki, M. Meyer, K. Puntener, S. Raab, A. Ruf, D. Schlatter, P. Mohr, P. *Bioorg. Med. Chem. Lett.* **2009,** *19,* 2468-2473.
44. N. Mahindroo, Y. H. Peng, C. H. Lin, U. K. Tan, E. Prakash, T. W. Lien, I. L. Lu, H. J. Lee, J. T. A. Hsu, X. Chen, C. C. Liao, P. C. Lyu, Y. S. Chao, S. Y. Wu, H. P. Hsieh, *J. Med. Chem.* **2006,** *49,* 6421-6424.
45. E. J. Levin, D. A. Kondrashov, G. E. Wesenberg, G. N. Phillips, *Structure*, **2007,** *15,* 1040-1052.
46. R. Epple, M. Azimioara, R. Russo, Y. Xie, X. Wang, C. Cow, J. Wityak, D. Karanewsky, B. Bursulaya, A. Kreusch, T. Tuntland, A. Gerken, M. Iskandar, E. Saez, H. M. Seidel, S. S. Tian, *Bioorg. Med. Chem. Lett.* **2006,** *16,* 5488-5492.
47. I. Pettersson, S. Ebdrup, M. Havranek, P. Pihera, M. Korinek, J. P. Mogensen, C. B. Jeppesen, E. Johansson, P. Sauerberg, *Bioorg. Med. Chem. Lett.* **2007,** *17,* 4625-4629.
